# Microwave Assisted Synthesis of 2,2′-Arylene-substituted Bis(4*H*-3,1-Benzoxazin-4-one) Derivatives Using the Complex Cyanuric Chloride/*N*,*N*-Dimethylformamide

**DOI:** 10.3390/molecules171011607

**Published:** 2012-09-28

**Authors:** Mehdi Shariat, Mohd Wahid Samsudin, Zuriati Zakaria

**Affiliations:** 1 School of Chemical Science and Food Technology, Faculty of Science and Technology, University Kebangsaan Malaysia (UKM), Kuala Lumpur 43600, Malaysia; 2 Department of Chemistry, Payame Noor University of Golpayegan, Isfahan 19395-4697, I. R., Iran; 3 Malaysia-Japan international institute of Technology, University Technology Malaysia (UTM), Kuala Lumpur 54100, Malaysia

**Keywords:** bis(4*H*-3,1-benzoxazin-4-one), OLED, microwave, UV

## Abstract

A new and efficient method has been designed to prepare 2,2′-arylene-substituted bis(4*H*-3,1-benzoxazin-4-one) derivatives by using the mixture of cyanuric chloride and *N*,*N*-dimethylformamide in a microwave-assisted reaction. The method used and presented here has good rate enhancement and excellent yields.

## 1. Introduction

Due to their interesting characteristics, 2,2′-arylene-bis(4*H*-3,1-benzoxazin-4-one) derivatives are an important class of condensed heterocyclic compounds. They are used as starting materials for preparation of different industrial products. Some are used for producing organic light emitting devices (OLEDs) [1,2,3,4].

**Figure 1:**
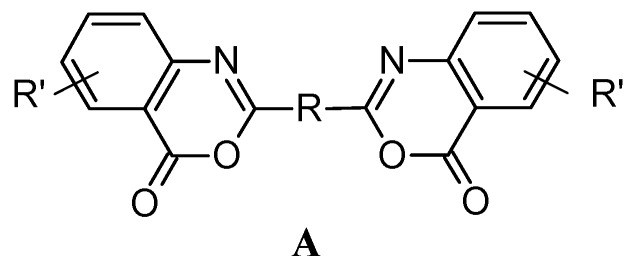
2,2′-arylene-bis(4*H*-3,1-benzoxazin-4-one) derivatives.

An OLED is an organic electroluminescent device in which light emission is produced in response to an electric current. OLED is the main part of notebooks, flat-panel displays, television screens, instrumentation panels and computer displays [3,4]. Some of these devices have a layer of organic compounds, as shown in formula **A** (Figure 1), situated between two electrodes (R: phenylene, naphthylene or biphenylene, R′: aryl or alkyl).

For example, 2,2′-(1,3-phenylene)bis(4*H*-3,1-benzoxazin-4-one) and 2,2′-(4,4′-biphenylene) bis(4*H*-3,1-benzoxazin-4-one) are used as an organic layer in OLEDs [4]. In addition, some of these compounds are strong broadband UV absorbers. They are used as additives in polymer chemistry. For instance, 2,2′-(1,4-phenylene)bis(4*H*-3,1-benzoxazin-4-one) was used in high-temperature processing of polycarbonate and polyethylene terephthalate because of its heat stability and low interaction with metals and also because of its UV absorption properties [5].

The most reported methods for synthesis of 2-substituted-4*H*-3,1-benzoxazin-4-one derivatives are those based on preparation of an acid-amide intermediate that is cyclized by various cyclizing agents such as acetic anhydride [6,7], polyphosphoric acid [8] triphenyl phosphite, pyridine [9], and cyanuric chloride [10,11]. Formation of the acid-amide intermediate is a key step of these methods.

**Figure 2:**
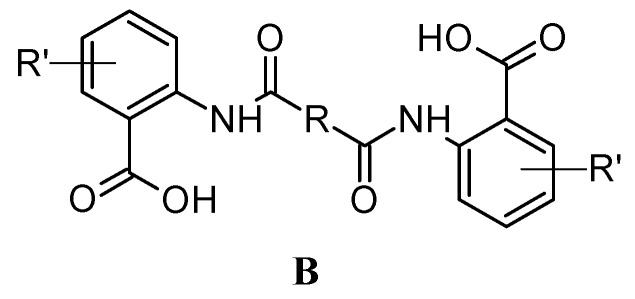
Acid-amide intermediate from anthranilic acid derivatives and a dichloride of a dicarboxylic acid.

The reported methods for producing 2,2′-arylene-bis(4*H*-3,1-benzoxazin-4-one) compounds **A** are based on the preparation of molecules **B** (Figure 2) as an intermediate from anthranilic acid derivatives and a dichloride of a dicarboxylic acid [1,2,3,4].

Because of the individual structure of molecules **B**, they can react with organic or inorganic Lewis acids in addition-substitution reactions. The result is the conversion of COOH group to an active ester. In other words, the OH group becomes a better leaving group.

The reaction product of cyanuric chloride (CC) and *N,N*-dimethylformamide (DMF) acts as an organic Lewis acid in a number of common reactions. For example, it is used for conversion of a wide series of secondary and primary alcohols to the matching alkyl chlorides and iodides [12,13]. Further more, CC/DMF is used for the conversion of *β*-amino alcohols into the corresponding chlorides [13]. In addition, a variety of ketoximes prepared from the related ketones, undergo the Beckmann rearrangement upon reaction with a mixture of CC/DMF [14].

**Scheme 1:**
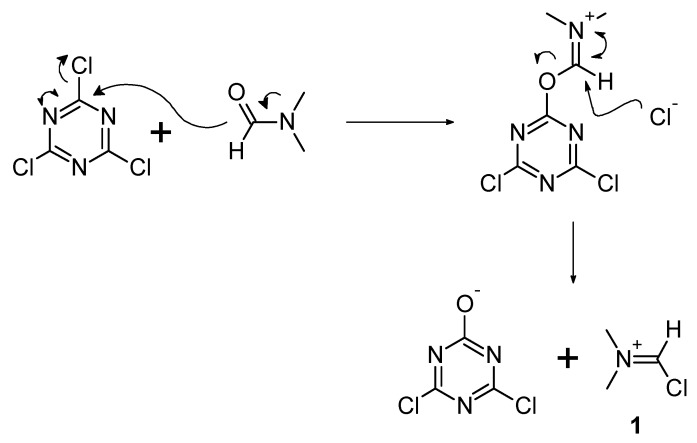
Preparation of iminium cation **1** from CC and DMF.

Cyanuric chloride reacts with *N,N*-dimethylformamide and iminium cation **1** is the result. During the reaction, cyanuric chloride is changed into hydroxydichloro-[1,3,5]-triazine [13]. The supposed mechanism is drawn below (Scheme 1).

Due to the nature of the mixture of cyanuric chloride and *N,N*-dimethylformamide, it should be an excellent reagent for microwave assisted reactions because of the ionic solution of the mixture and good dielectric constant of DMF. The electric constituent of an electromagnetic wave at microwave frequency causes heating by two main mechanisms: ionic conduction and dipolar polarization [15].

## 2. Results and Discussion

**Scheme 2:**
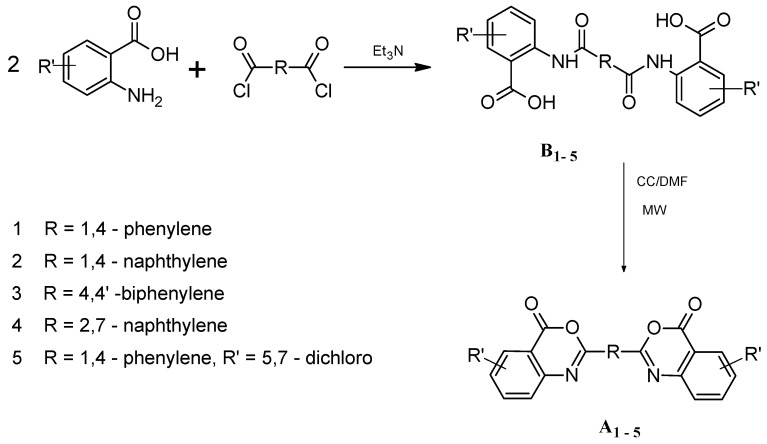
The reaction pathway from anthranilic acid derivatives to 2,2′-arylene-bis(4*H*-3,1-benzoxazin-4-one) derivatives.

The intermediates **B_1–5_** can be prepared from anthranilic acid derivatives and dihalides of an aromatic dicarboxylic acid, in refluxing neutral organic solvent in the presence of triethylamine, which produces more than 85% yield (Scheme 2).

In the second step, by using the mixture of cyanuric chloride and *N,N*-dimethylformamide in a microwave-assisted cyclodehydration reaction, 2,2′-arylene-bis(4*H*-3,1-benzoxazin-4-one) derivatives **A_1–5_** were prepared in more than 70% yield (Table 1).

**Scheme 3:**
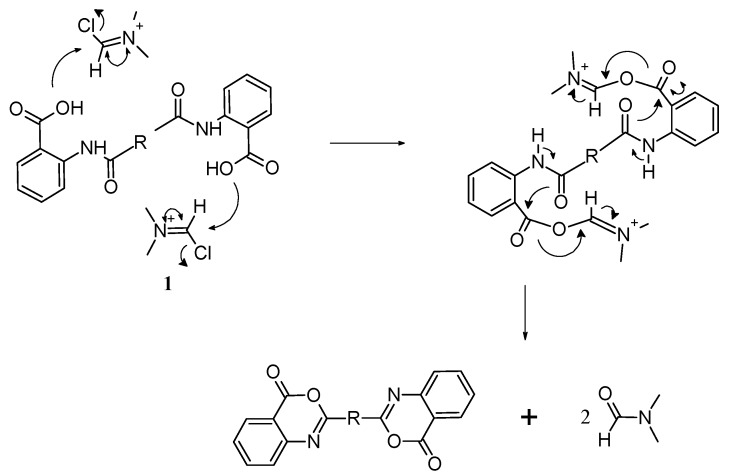
Benzoxazinone ring formation from acid-amid intermediate and iminium cation.

In view of the structural characteristics of intermediates formula **B**, the iminium cation **1** from CC/DMF can act as a cyclizing agent by conversion of the carboxylic group into an active ester. The proposed mechanism is drawn in Scheme 3. DMF catalyzes the reaction and acts as a solvent too.

In this case the temperature was increased unexpectedly because of the ionic nature of the mixture. In this method, the reaction time is shorter than in previous methods and the reaction is very clean. In addition, the product can be isolated with high purity by a very simple workup.

## 3. Experimental 

### 3.1. General

The structures of products **A_1–5_** and **B_1–5_** were confirmed by analysis using spectra data (FT-IR, ^1^H-NMR, ^13^C-NMR, UV and MS). All machines, equipments and instruments were used at analytical laboratories of faculty of science and technology (PPSKTM, FST), University Kebangsaan Malaysia (UKM). The FT-IR spectra were measured on a Perkin Elmer Model GX spectrometer, using KBr pellets. The NMR spectra were recorded on a Bruker/AVANCE III 600 MHz spectrometer and chemical shifts are reported relative to TMS. The mass spectra (MS-MS) were recorded using a Dionex/Bruker micro TOF-Q instrument. The elemental analysis (CHN) was recorded by Thermo Finngan instrument. The melting points were measured in open capillary tubes. The UV absorption was measured by a Shimadzu UV-2400PC series instrument. All commercial reagents were used as received without additional purification. The procedures for preparation and purification of reported products are the same.

### 3.2. Preparation of 2,2′-(1,4-Phenylene)bis(4H-3,1-benzoxazin-4-one) *(**A_1_**)*

#### 3.2.1. Step 1: Preparation of 2,2′-[1,4-Phenylene-bis(carbonylimino)]dibenzoic Acid

A solution of terephthaloyl chloride (0.609 g, 3 mmol) in chloroform (5 mL) was added to a stirred solution of anthranilic acid (0.823 g, 6 mmol) and triethylamine (0.85 mL, 6.1 mmol) in chloroform (15 mL). The mixture was stirred at rt. for 4 h, then, the solvent was evaporated under vacuum, and the precipitate was washed with dilute acid (HCl 1%, 10 mL) and distilled water (two times, 15 mL each). After drying the product, it was recrystallized from a 1:3 *N*-methylpyrrolidone/ethanol mixture to give 2,2′-[1,4-phenylene-bis(carbonylimino)]dibenzoic acid (**B_1_**, 1.19 g, 98%). M.p. 324–326 °C. IR (KBr): *v*_max_/cm^−1^ 2468–3113, 1701, 1663, 1610, 1546. ^1^H-NMR (DMSO): δ ppm 12.54 (s, 2H, COOH), 8.17 (s, 2H, NHCO), 8.05 (s, 4H, Ar–H), 7.19–8.14 (m, 8H, Ar–H). HRMS (MS-MS): *m/z* 403.0934 [M−H]^−^, Calcd. for C_22_H_16_N_2_O_6_. Anal. Calcd. for C_22_H_16_N_2_O_6_ (404.3722): C, 65.34; H, 3.99; N, 6.93. Found: C, 65.37; H, 3.93; N, 6.89.

#### 3.2.2. Step 2: Reaction of 2,2′-[1,4-Phenylene-bis(carbonylimino)]dibenzoic Acid with CC/DMF

Cyanuric chloride (0.552 g, 3 mmol) in DMF (5 mL) was added to a stirred mixture of intermediate **B_1_** (0.607 g, 1.5 mmol) in DMF (10 mL) in a 25 mL vial. Then, microwave irradiation (350 W power) was applied for 1 min. The mixture was poured in distilled water (20 mL) and ice. Then, the solids were washed with a saturated solution of NaHCO_3_ (10 mL) and distilled water (two times, 15 mL each). The yellowish precipitate was recrystallized from a 2:3 NMP/ethanol mixture to give the product **A_1_** (0.486 g 88%). M.p. 315–316 °C; lit. 315 °C [16]; IR (KBr): *v*_max_/cm^−1^ 1759, 1620, 1596. ^1^H-NMR (DMSO): δ ppm 7.65–8.40 (m, 8H, Ar–H), 8.23 (s, 4H, Ar–H). ^13^C-NMR (DMSO): 159.4 (C=O); 156.9 (C_2_, C_2_′); 146.7 (C_8a_, C_8a_′); 137.3 (C_7_, C_7_′); 133.2 (C_1_, C_4_ phenyl ring); 130.5 (C_5_, C_5_′); 129.5 (C_2_, C_3_, C_5_, C_6_ phenyl ring); 128.30 (C_6_, C_6_′); 127.4 (C_8_, C_8_′); 117.5 (4_a_, 4_a_′). HRMS (MS-MS): *m/z* 369.0711 [M+H]^+^, Calcd. for C_22_H_12_N_2_O_4_. Anal. Calcd. for C_22_H_12_N_2_O_4_ (368.3416): C, 71.74; H, 3.28; N, 7.61. Found: C, 71.77; H, 3.26; N, 7.59. UV: λ (nm) 305, 261.

### 3.3. Preparation of 2,2′-(1,4-Naphthylene)bis(4H-3,1-benzoxazin-4-one) *(**A_2_**)*

#### 3.3.1. Step 1: Preparation of 2,2′-[1,4-Naphthylene-bis (carbonylimino)] Dibenzoic Acid

Naphthalene-1,4-dicarbonyl dichloride and anthranilic acid were used for preparation of 2,2′-[1,4-naphthylene-bis (carbonylimino)] dibenzoic acid **B_2_** using the same procedure as for **B_1_** (yield: 95%; m.p. 256–258 °C). IR (KBr): *v*_max_/cm^−1^ 2560–3198, 1656, 1604, 1526. ^1^H-NMR (DMSO): δ ppm 11.84 (s, 2H, COOH), 8.05 (s, 2H, NHCO), 7.98 (s, 2H, Ar–H), 7.28–8.65 (m, 12H, Ar–H). HRMS (MS-MS): *m/z* 453.1146 [M−H]^−^, Calcd. for C_26_H_18_N_2_O_6_. Anal. Calcd. for C_26_H_18_N_2_O_6_ (454.4309): C, 68.73; H, 3.99; N, 6.16. Found: C, 68.76; H, 3.98; N, 6.19.

#### 3.3.2. Step 2: Reaction of 2,2′-[1,4-Naphthylene-bis (carbonylimino)] Dibenzoic Acid with CC/DMF

2,2′-(1,4-Naphthylene)-bis-(4*H*-3,1-benzoxazin-4-one) **A_2_** was prepared by using MW (350 W) for 1.5 min. The procedure was the same as procedure **A_1_** (yield: 85%). M.p. 268–270 °C; IR (KBr): *v*_max_/cm^−1^ 1774, 1766, 1604; ^1^H-NMR (DMSO): δ ppm 8.31 (s, 2H, Ar–H), 7.36–8.63 (m, 12H, Ar–H). ^13^C-NMR (DMSO):159.6 (C=O); 157.2 (C_2_, C_2_′); 146.5 (C_8a_, C_8a_′); 137.3 (C_7_, C_7_′); 131.8 (C_4a_, C_8a_ naphthalene ring); 130.9 (C_1_, C_4_ naphthalene ring); 129.7 (C_5_, C_5_′); 128.8 (C_8_, C_5_ naphthalene ring); 128.5 (C_6_, C_6_′); 128.3 (C_6_, C_7_ naphthalene ring); 127.7 (C_2_, C_3_ naphthalene ring); 126.8 (C_8_, C_8_′); 117.8 (C_4a_, C_4a_′). HRMS (MS-MS): *m/z* 419.1029 [M+H]^+^, Calcd. for C_26_H_14_N_2_O_4_. Anal. Calcd. for C_26_H_14_N_2_O_4_ (418.0953): C, 74.64; H, 3.37; N, 6.70. Found: C, 74.76; H, 3.34; N, 6.73. UV: λ (nm) 305, 261.

### 3.4. Preparation of 2,2′-(4,4′-Biphenylene)bis(4H-3,1-benzoxazin-4-one) *(**A_3_**)*

#### 3.4.1. Step 1: Preparation of 2,2′-[4,4′-Biphenylene-bis(carbonylimino)]dibenzoic Acid

2,2′-[4,4′-Biphenylene-bis(carbonylimino)]dibenzoic acid **B_3_** was prepared by using the method of **B_1_** (yield: 97%). M.p. 286–288 °C. IR (KBr): *v*_max_/cm^−1^ 2768–3129, 1652, 1606, 1537, 1509. ^1^H-NMR (DMSO): δ ppm 12.25 (s, 2H, COOH), 8.08 (s, 2H, NHCO), 7.85–8.73 (m, 16H, Ar–H). HRMS (MS-MS): *m/z* 479.1235 [M−H]^−^, Calcd. for C_28_H_20_N_2_O_6_. Anal. Calcd. for C_28_H_20_N_2_O_6_ (480.4682): C, 69.99; H, 4.20; N, 5.83. Found: C, 70.03; H, 4.28; N, 5.79.

#### 3.4.2. Step 2: Reaction of 2,2′-[4,4′-Biphenylene-bis(carbonylimino)]dibenzoic Acid with CC/DMF

The procedure was the same as for **A_2_**, and 2,2′-(4,4′-biphenylene)-bis-(4*H*-3,1-benzoxazin-4-one) **A_3_** was prepared in 85% yield. M.p. 376–378 °C. IR (KBr): *v*_max_/cm^−1^ 1741, 1679, 1553. ^1^H-NMR (DMSO): δ ppm 7.26–8.45 (m, 16H, Ar–H). ^13^C-NMR (DMSO): 159.4 (C=O); 156.6 (C_2_, C_2_′); 146.8 (C_8a_, C_8a_′); 137.4 (C_1_, C_1_′ biphenylene ring); 131.8 (C_7_, C_7_′); 130.2 (C_5_, C_5_′); 129.2 (C_5_, C_5_′ biphenylene ring); 128.6 (C_4_, C_4_′ biphenylene ring); 128.0 (C_2_, C_6_, C_2_′, C_6_′ biphenylene ring); 127.9 (C_6_, C_6_′); 127.5 (C_8_, C_8_′); 117.6 (C_4a_, C_4a_′). HRMS (MS-MS): *m/z* 445.1015 [M+H]^+^, Calcd. for C_28_H_16_N_2_O_4_. Anal. Calcd. for C_28_H_16_N_2_O_4_ (444.4376): C, 75.63; H, 3.63; N, 6.30. Found: C, 75.71; H, 3.68; N, 6.33. UV: λ (nm) 340.

### 3.5. Preparation of 2,2′-(2,7-Naphthylene)bis(4H-3,1-benzoxazin-4-one) *(**A4**)*

#### 3.5.1. Step1: Preparation of 2,2′-[2,7-Naphthylene-bis(carbonylimino)]dibenzoic Acid

For 2,2′-[2,7-naphthylene-bis(carbonylimino)]dibenzoic acid **B_4_**, naphthylene-2,7-dicarbonyl dichloride and anthranilic acid were used (Yield: 96%; m.p. 274–276 °C). IR (KBr): *v*_max_/cm^−1^ 2875–3119, 1665, 1608, 1589, 1537. ^1^H-NMR (DMSO): δ ppm 12.36 (s, 2H, COOH), 8.24 (s, 2H, NHCO), 7.25–8.74 (m, 14H, Ar–H). HRMS (MS-MS): *m/z* 453.1087 [M−H]^−^, Calcd. for C_26_H_18_N_2_O_6_. Anal. Calcd. for C_26_H_18_N_2_O_6_ (454.4309): C, 68.73; H, 3.99; N, 6.16. Found: C, 68.80; H, 3.93; N, 6.20.

#### 3.5.2. Step 2: Reaction of 2,2′-[2,7-Naphthylene-bis(carbonylimino)]dibenzoic Acid with CC/DMF

The procedure of the second step was the same as for **A_1_**. Microwave irradiation was used for 2 min to give 2,2′-(2,7-naphthylene)-bis-(4*H*-3,1-benzoxazin-4-one) (**A_4_**, yield: 79%; m.p. 278–280 °C). IR (KBr): *v*_max_/cm^−1^ 1767, 1720, 1569. ^1^H-NMR (DMSO): δ ppm 7.27–8.50 (m, 14H, Ar–H). ^13^C-NMR (DMSO): 159.7 (C=O); 156.8 (C_2_, C_2_′); 146.3 (C_8a_, C_8a_′); 137.8 (C_4a_ naphthalene ring); 136.7 (C_7_, C_7_′); 133.8 (C_8a_ naphthalene ring); 131.5 (C_5_, C_5_′); 128.8 (C_2_, C_7_ naphthalene ring); 128.2 (C_4_, C_5_ naphthalene ring); 127.9 (C_1_, C_8_ naphthalene ring); 127.3 (C_6_, C_6_′); 127.1 (C_3_, C_6_ naphthalene ring); 121.8 (C_8_, C_8_′); 116.8 (C_4a_, C_4a_′). HRMS (MS-MS): *m/z* 419.1000 [M+H]^+^, Calcd. for C_26_H_14_N_2_O_4_. Anal. Calcd. for C_26_H_14_N_2_O_4_ (418.4003): C, 74.64; H, 3.37; N, 6.70. Found: C, 74.57; H, 3.36; N, 6.76. UV: λ (nm) 307, 394.

### 3.6. Preparation of 2,2′-(1,4-Phenylene)bis(5,7-dichloro-4H-3,1-benzoxazin-4-one) *(**A_5_**)*

#### 3.6.1. Step 1: Preparation of 2,2′-[1,4-Phenylene-bis (carbonylimino)]bis(4,6-dichlorobenzoic acid)

The reaction between terephthaloyl chloride and 2-amino-4,6-dichlorobenzoic acid was used to give 2,2′-[1,4-phenylene-bis (carbonylimino)]bis(4,6-dichlorobenzoic acid) (**B_5_**) using the same procedure as for **B_1_**; yield: 95%; m.p. 298–300 °C). IR (KBr): *v*_max_/cm^−1^ 2668–3157, 1739, 1690, 1565. ^1^H-NMR (DMSO): δ ppm 10.69 (s, 2H, COOH), 8.05 (s, 2H, NHCO), 8.02 (s, 4H, Ar–H), 7.90 (d, 2H; H_3_, H_3′_, *J* = 1.80 Hz), 7.80 (d, 2H; H_5_, H_5′_, *J* = 1.82 Hz). HRMS (MS-MS): *m/z* 538.9382 [M−H]^−^, Calcd. for C_22_H_12_Cl_4_N_2_O_6_. Anal. Calcd. for C_22_H_12_Cl_4_N_2_O_6_ (542.1524): C, 48.74; H, 2.23; N, 5.17. Found: C, 48.81; H, 2.20; N, 5.11.

#### 3.6.2. Step 2: Reaction of 2,2′-[1,4-Phenylene-bis (carbonylimino)]bis(4,6-dichlorobenzoic acid) with CC/DMF

The procedure was the same as for **A_4_** (yield: 77%). M.p. 325–327 °C. IR (KBr): *v*_max_/cm^−1^ 1766, 1619, 1582. ^1^H-NMR (DMSO): δ ppm 8.03 (s, 4H, Ar–H), 7.82 (d, 2H; H_6_, H_6_′ *J* = 1.81 Hz), 7.84 (d, 2H; H_8_, H_8′_ *J* = 1.78 Hz). ^13^C-NMR (DMSO): 159.5 (C=O); 156.3 (C_2_, C_2_′); 146.6 (C_8a_, C_8a_′); 139.4 (C_7_, C_7_′); 135.7(C_5_, C_5_′); 131.9 (C_1_, C_4_ phenyl ring); 128.6 (C_2_, C_3_, C_5_, C_6_ phenyl ring); 128.2 (C_6_, C_6_′); 120.6 (C_4a_, C_4a_′); 119.43 (C_8_, C_8_′). HRMS (MS-MS): *m/z* 526.5189 [M+Na]^+^, Calcd. for C_22_H_8_Cl_4_N_2_O_4_. Anal. Calcd. for C_22_H_8_Cl_4_N_2_O_4_ (506.1219): C, 52.21; H, 1.59; N, 5.53. Found: C, 52.24; H, 1.58; N, 5.51. UV: λ (nm) 311, 262.

## 4. Conclusions 

We have found that a mixture of CC/DMF is an effective reagent in the microwave-assisted synthesis of 2,2′-(arylene)bis(4*H*-3,1-benzoxazin-4-one) derivatives. Furthermore, in this method, the role of solvent is interesting because the solvent also catalyzed the reaction. The method should be applicable for the synthesis of other 2,2′-arylene-substituted bis(4*H*-3,1-benzoxazin-4-one) derivatives. The short reaction times, the availability and simplicity of the starting materials, simple workup and the experimental procedure make this new path more efficient than previous methods.

## Figures and Tables

**Table 1:** Yield, Time of Reaction (Power 350 W) and Melting Point.

Name and Structure	Product	Yield (%)	Time (min)	M.p. (°C)
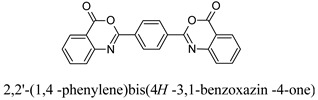	A_1_	88	1	314–316
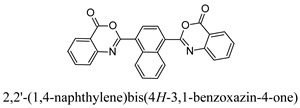	A_2_	85	1.5	268–270
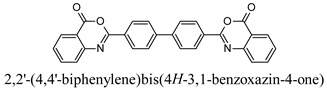	A_3_	85	1.5	376–378
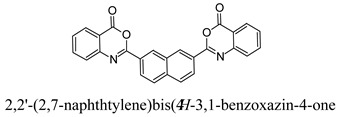	A_4_	79	2	278–280
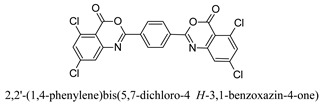	A_5_	77	2	325–327
